# Identification of predictive biomarkers for nivolumab efficacy in non-small cell lung cancer through integrated serum lipidomics and proteomics analysis

**DOI:** 10.3389/fimmu.2026.1773700

**Published:** 2026-04-14

**Authors:** Hiroaki Hase, Yoshito Takeda, Shohei Koyama, Yujiro Naito, Kentaro Jingushi, So-Ichiro Fukada, Kazutake Tsujikawa

**Affiliations:** 1 Laboratory of Stem Cell Regeneration and Adaptation, Graduate School of Pharmaceutical Sciences, Osaka University, Osaka, Japan; 2 Department of Respiratory Medicine and Clinical Immunology, Graduate School of Medicine, Osaka University, Osaka, Japan; 3 Laboratory of Molecular and Cellular Physiology, Graduate School of Pharmaceutical Sciences, Osaka University, Suita, Osaka, Japan

**Keywords:** biomarker, immune checkpoint inhibitors, non-small cell lung cancer, phospholipid, proteome

## Abstract

Immune checkpoint inhibitors (ICIs) have revolutionized the treatment of non-small cell lung cancer (NSCLC); however, their efficacy is confined to a subset of patients. The urgent development of biomarkers capable of predicting therapeutic efficacy prior to treatment is essential, as this could mitigate unnecessary adverse effects and reduce healthcare costs. In this study, we conducted an integrated lipidomic (phospholipid) and proteomic (whole serum and extracellular vesicle) analysis of pre-treatment serum samples obtained from patients with NSCLC who received nivolumab. The pre-treatment serum phospholipid profiles revealed significant differences between responder and non-responder groups. Notably, the lysophosphatidylcholine (LPC) class—particularly LPC(20:0)—emerged as a predictive biomarker, exhibiting elevated levels in responders (AUC = 0.782; LOOCV AUC = 0.720; Bootstrap AUC = 0.781). Proteomic analysis further indicated increased expression of complement components and acute-phase proteins in the non-responder group. Moreover, integration of serum, extracellular vesicle proteome, and phospholipid datasets using Weighted Gene Co-expression Network Analysis and Multi-Omics Factor Analysis suggested that biological processes potentially associated with LPC involve neutrophil and platelet activation pathways. Pre-treatment serum LPC levels are a promising biomarker for predicting the response to nivolumab therapy in NSCLC. This LPC signature reflects a systemic immunometabolic state involving platelet and neutrophil activity, suggesting a novel biological mechanism underlying ICI treatment efficacy.

## 1 Introduction

Immune checkpoint inhibitors (ICIs) have revolutionized the therapeutic approach to non-small-cell lung cancer (NSCLC). Nivolumab, an antibody targeting PD-1, enhances overall survival across histological subtypes with a favorable safety profile and, in certain cohorts, achieves long-term survival plateaus (1–3). Nevertheless, durable benefit is limited to a clinically meaningful subset of patients. Primary resistance remains frequent, and immune-related adverse events (irAEs) can be induced (4–9). These realities underscore the urgent need for pre-treatment biomarkers that (i) are enriched for patients likely to benefit, (ii) minimize futile exposure and toxicity, and (iii) guide rational combinations or sequencing with chemotherapy, molecularly targeted agents, or other ICIs.

Currently approved biomarkers for predicting ICI response include PD-L1 expression and tumor mutational burden (TMB). PD-L1 is the most widely used predictive biomarker; however, responses are observed across all expression levels, and a substantial proportion of PD-L1-high patients do not respond to therapy (10–13). TMB has also shown promise as a tissue-based biomarker, but its clinical utility is limited by the lack of harmonized cutoff values and its inability to capture the host immune status. These tumor-intrinsic markers do not reflect the systemic immunological context of the host, which may be equally critical for determining ICI responsiveness. This gap motivates the search for circulating biomarkers that capture host immune competence independent of tumor characteristics. Liquid biopsy modalities—including circulating cell-free DNA, extracellular vesicles, and soluble proteins—extend biomarker accessibility to the systemic compartment (14, 15). Among emerging liquid biopsy approaches, lipidomic profiling is particularly compelling because it provides information orthogonal to nucleic acid- or protein-based biomarkers, offering a means to characterize systemic immunometabolic programs that shape the host’s capacity to respond to immunotherapy.

The importance of lipid metabolism extends far beyond its traditional role in membrane structure. Lipids are now understood as active regulators of immune cell function at multiple levels. At the membrane level, phospholipids control the dynamics of lipid rafts and receptor clustering at the immune synapse, while their phospholipase-mediated remodeling generates diverse lipid mediators (16). Sphingolipids set thresholds for apoptosis and cytokine receptor signaling, while oxidized phospholipids function as damage-associated molecular patterns that trigger innate immune responses. Lysophospholipids represent another critical class, regulating T cell activation, myeloid cell polarization, neutrophil behavior, and dendritic cell function (17, 18). These pathways do not act independently of cancer biology. Tumor cells and the surrounding stroma actively utilize lipid synthesis and remodeling to create an immunosuppressive microenvironment that restricts T cell function and promotes immune evasion. Importantly, these metabolic alterations are not confined to the tumor site. They propagate into the systemic circulation, potentially leaving detectable traces in the serum phospholipidome that reflect both local tumor activity and the host’s broader immunometabolic state. This systemic reflection of tumor–immune interplay represents an information dimension difficult to capture with nucleic acid– or protein-based biomarkers. Despite this compelling biological basis, the predictive value of pre-treatment circulating phospholipid profiles for immune checkpoint inhibitor therapy in NSCLC remains largely unexplored.

In this study, we examined whether pre-treatment serum phospholipids are associated with nivolumab response using a semi-targeted lipidomic workflow. To enhance biological interpretability across heterogeneous clinical and pathological contexts, we integrated complementary proteomic layers—whole serum and extracellular vesicle (EV) proteomes— allowing lipid-associated signals to be interpreted within relevant immunological networks. This design prioritizes the identification of candidate lipid biomarkers and situates them within biologically coherent networks, thereby generating hypothesis-driven insights into pathways associated with clinical outcomes.

## 2 Materials and methods

### 2.1 Study design and patient cohort

This study was conducted as part of a multi-institutional observational research project approved by the Ethics Committee of Osaka University Hospital (Approval No.: 17148-6) and the Ethics Committee of the Graduate School of Pharmaceutical Sciences Osaka University (Approval No.: 2023-8). Written informed consent was obtained from all participants or their legally authorized representatives, in accordance with the Declaration of Helsinki. Clinical information and biospecimens were anonymized using a linkable code system before analysis. Patients diagnosed with NSCLC who subsequently received nivolumab therapy were prospectively enrolled. Peripheral blood was obtained before the first administration of nivolumab. For each participant, peripheral blood was collected in serum-separation tubes during routine clinical blood sampling to minimize patient burden. After coagulation, samples were centrifuged to isolate serum and stored at −80 °C until use.

#### 2.1.1 Sample preparation

Serum samples were processed separately for each type of mass spectrometric analysis. For phospholipid analysis, three volumes of methanol were added to the serum, followed by centrifugation at 16,400 × g for 20 min to precipitate proteins. The resulting supernatant was diluted tenfold with 50% methanol and analyzed. For serum proteome analysis, major serum proteins such as albumin and immunoglobulins were depleted using the Proteome Purify™ 2 Human Serum Protein Immunodepletion Resin (R&D Systems, Inc.) according to the manufacturer’s instructions. EVs were isolated from serum using the MagCapture™ Exosome Isolation Kit PS Ver.2 (Fujifilm Wako Pure Chemical Corporation, Japan) following the manufacturer’s protocol. For proteomic analysis of serum and EVs, 100 µL of sample solution was first prepared, followed by the addition of 5 µL of 200 mM tris(2-carboxyethyl)phosphine (Thermo Fisher Scientific) and incubation at 55 °C for 1 h to reduce the disulfide bonds. Subsequently, 5 µL of 375 mM iodoacetamide (Thermo Fisher Scientific) was added and incubated at room temperature for 30 min for alkylation. Subsequently, 400 µL of methanol (Fujifilm), 100 µL of chloroform (Fujifilm), and 300 µL of water were added, vortex-mixed, and centrifuged at 14,000 × g for 3 min. The upper phase (approximately 80%) was discarded, and 300 µL of methanol was added to the remaining precipitate, followed by mixing and centrifugation at 14,000 × g for 4 min. The supernatant was removed, and the pellet was air-dried. The dried pellet was re-dissolved in 50 mM ammonium bicarbonate and digested overnight at 37 °C with 2.5 µg of trypsin (Thermo Fisher Scientific).

#### 2.1.2 Phospholipid analysis

Pre-treated serum samples were analyzed using a Nexera X3 Ultra-high-performance liquid chromatography (UHPLC) system coupled with an LCMS-8060NX triple quadrupole mass spectrometer (Shimadzu Corporation, Kyoto, Japan). The UHPLC mobile phases, gradient conditions, and detection of individual phospholipid species were conducted using a semi-targeted LC/MS/MS approach based on the MRM Library for Phospholipid Profiling (Shimadzu Corporation). Peak alignment and peak height quantification were performed using Traverse MS software (Shimadzu Corporation).

### 2.2 Proteomic analysis

Serum proteome analysis was performed using an Easy-nLC 1200 nano-LC system coupled online to an Orbitrap Eclipse Tribrid mass spectrometer (Thermo Fisher Scientific). Peptides were separated on a C18 analytical column (75 μm × 12 cm, 3 μm; Nikkyo Technos) using a binary gradient of water/acetonitrile with 0.1% formic acid. Data were acquired in data-independent acquisition (DIA) mode with MS¹ scans over m/z 500–1100 and MS² fragmentation using higher-energy collisional dissociation (HCD, normalized energy 25%). Raw files were processed using DIA-NN (version 1.8.1) in library-free mode. EV proteomic analysis was performed using a Vanquish Neo UHPLC system coupled to an Orbitrap Astral mass spectrometer (Thermo Fisher Scientific). Peptides were separated on an EASY-Spray™ LC column (Thermo Fisher Scientific) using a binary gradient of water/acetonitrile with 0.1% formic acid. Data were acquired in DIA mode with MS¹ scans over m/z 380–980 and MS² fragmentation using HCD (normalized energy 25%). Raw files were processed using DIA-NN (version 1.9.1) in library-free mode.

### 2.3 Statistical and bioinformatics analysis

All samples within each omics platform were processed and analyzed in a single analytical batch to eliminate inter-batch effects as a source of technical variation. For phospholipid analysis, principal component analysis (PCA), hierarchical clustering, and receiver operating characteristic curve area under the curve (AUC-ROC) calculations were performed using peak intensity. For comparisons of individual lipid species, p-values from Student’s t-tests were reported. To assess the statistical significance of separation between groups, permutational multivariate analysis of variance (PERMANOVA; 999 permutations) was applied to all PCA analyses. These analyses were performed using MetaboAnalyst (version 6.0) (19). To address overfitting risk when using all 42 samples for biomarker discovery without a held-out validation set, we performed leave-one-out cross-validation (LOOCV) and.632 bootstrap resampling (B = 2,000). ROC curve analysis, LOOCV, and bootstrap resampling were performed using Python 3.13.5 (scikit-learn 1.6.1, numpy 2.1.3, pandas 2.2.3). AUC values derived from LOOCV and 95% confidence intervals from the bootstrap method are reported throughout the manuscript alongside standard AUC estimates. Quantitative proteomics data from DIA-NN were preprocessed using Perseus (version 2.0.9.0) (20). Proteins with missing values exceeding 50% across all samples were removed, and remaining missing values were imputed using a Gaussian distribution shifted to the lower tail of the intensity distribution. Preprocessed data underwent PCA and hierarchical clustering using MetaboAnalyst. Differential abundance analysis between Responders and Non-Responders was performed using the R package MS-DAP (v1.0.6) (21) with DIA-NN output. Intra-sample normalization (variance-stabilizing normalization, vsn) was followed by inter-sample normalization (modebetween_protein). The MS-EmpiRe model was selected, and proteins meeting the Benjamini–Hochberg FDR-adjusted q < 0.05 threshold were considered significantly differentially abundant. The minimum fold-change threshold was empirically determined via bootstrap resampling and set to log_2_FC > 0.372 for the serum proteome and > 0.386 for the EV proteome. Volcano plots were generated using the R package plotly. For Weighted Gene Co-expression Network Analysis (WGCNA), the pickSoftThreshold function (scale-free topology criterion R² ≥ 0.85) was used to individually select the soft-threshold exponent for each network. A soft-threshold exponent of β = 4 was selected for both the serum proteome (R² = 0.958) and the EV proteome (R² = 0.879). Additional parameters were as follows: TOM type = “unsigned”; module merge height cutoff = 0.25 (minimum inter-module eigengene correlation 0.75); minimum module size was set adaptively (serum: 10–30 proteins; EV: 10–20 proteins). LPC species levels were not included as WGCNA network input but were used as external traits for module–trait correlation analysis; modules significantly correlated with LPC levels were defined by |r| > 0.4 and BH-corrected p < 0.05. In MOFA2, input features underwent variance-based pre-filtering (top 100 for phospholipids; top 500 for each proteome layer) and autoscaling (mean-centering and division by standard deviation) within each layer. Multi-Omics Factor Analysis (MOFA2) was applied with K = 10 latent factors, convergence mode “medium” (ELBO criterion), a fixed random seed (seed = 2024), and a maximum of 500 iterations. Each omics layer was modeled as a separate view with independent noise parameters, accounting for differences in scale and data characteristics between modalities. Factor scores were compared between Responders and Non-Responders using the Wilcoxon rank-sum test with Benjamini–Hochberg correction. Enrichment analysis was performed using Metascape (22), and bar graphs were generated using Microsoft 365 Excel (Microsoft Corporation, Redmond, WA, USA).

## 3 Results

### 3.1 Pre-treatment serum phospholipid profiles distinguish responders from non-responders

**Figure 1:**
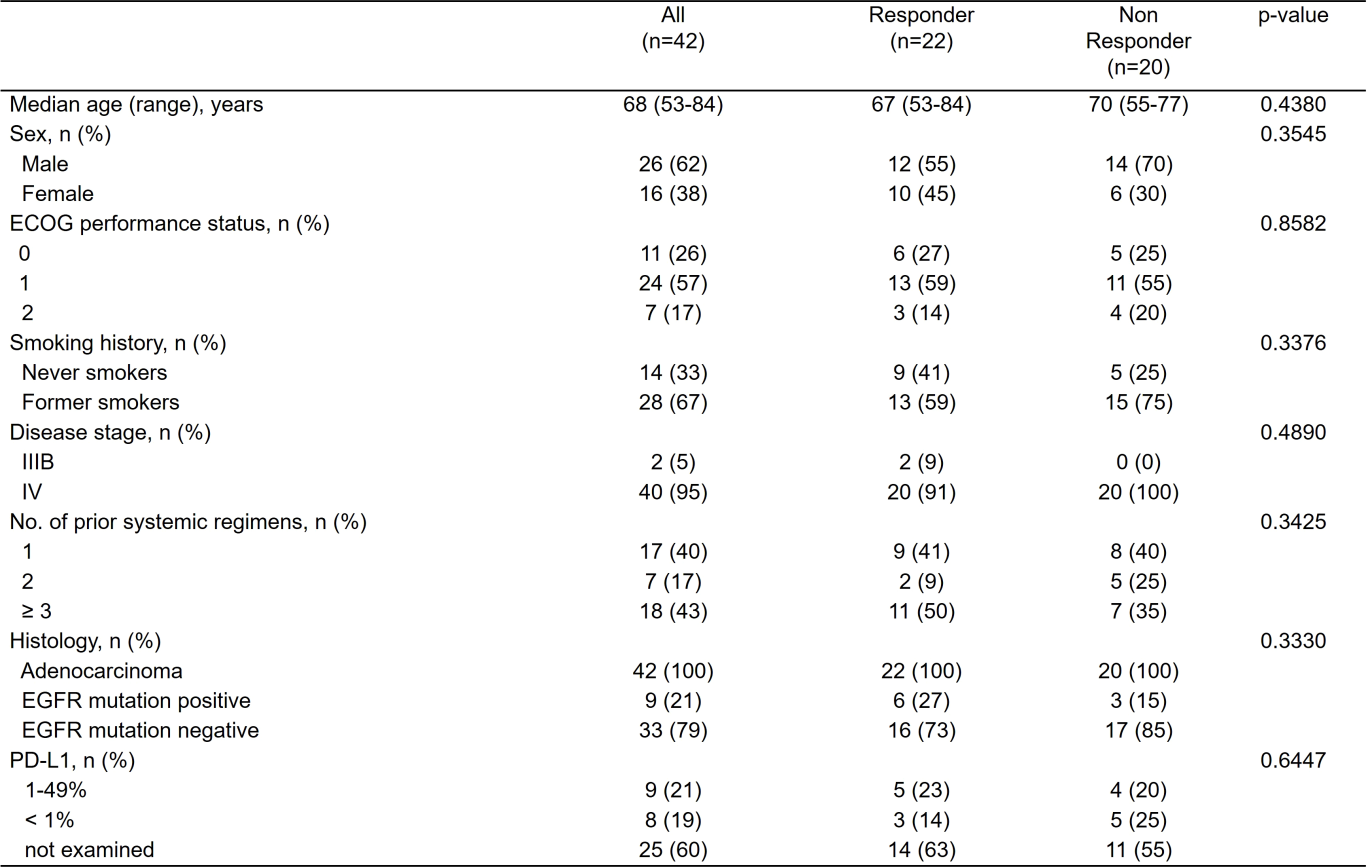
Table of patient characteristics at baseline (n=42).

**Figure 2:**
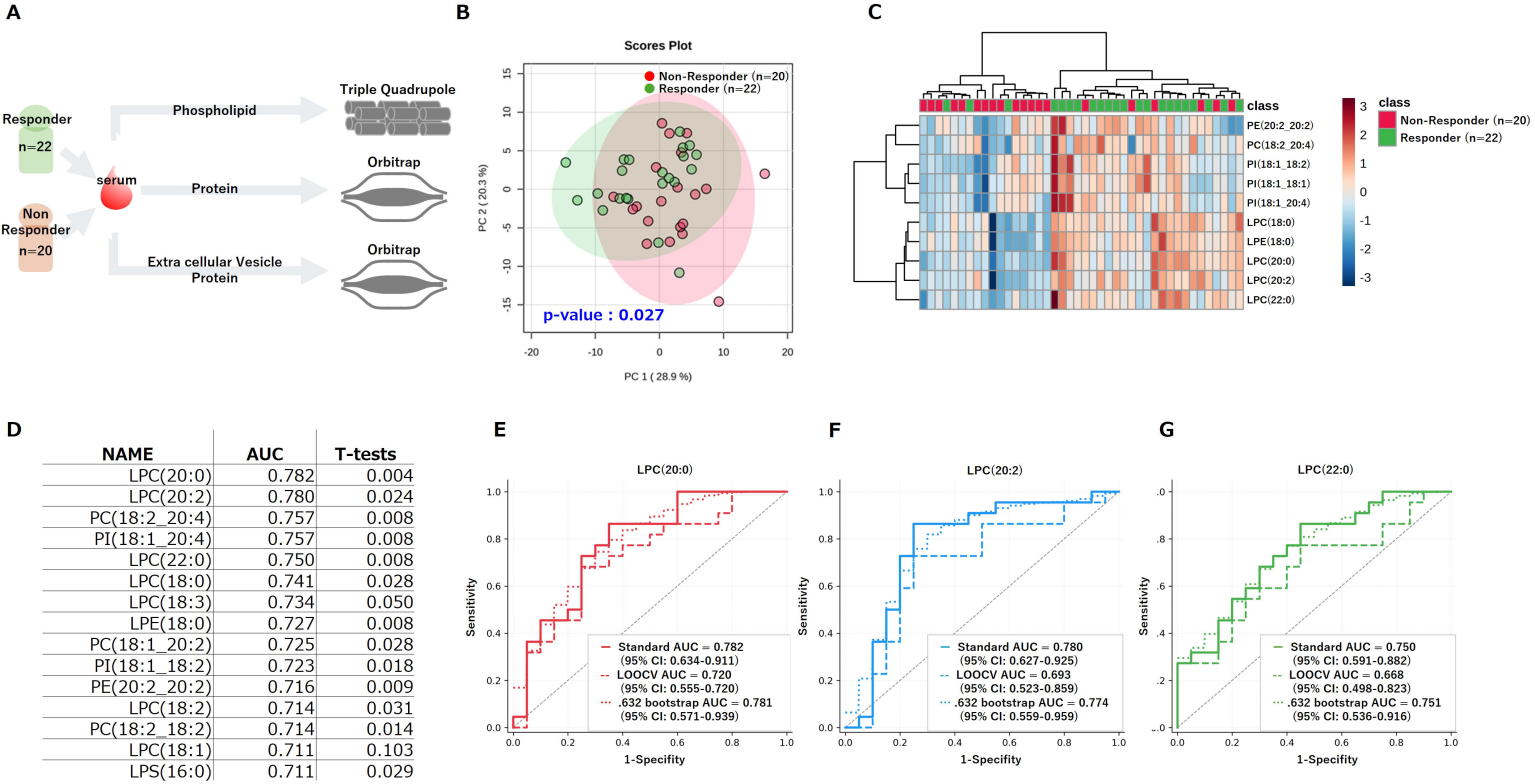
Pre-treatment serum phospholipid profiles stratify responders and non-responders to nivolumab. **(A)** Schematic diagram illustrating the experimental design of the study. (n = 22 Responders; n = 20 Non-Responders) **(B)** PCA plot based on pre-treatment serum phospholipid profiles, showing separation between Responders and Non-Responders (PERMANOVA p = 0.027). **(C)** Hierarchical clustering heatmap based on the most differentially abundant phospholipids, demonstrating that patient categorization corresponds with clinical response (Reponder/Non-Responder). **(D)** Table of univariate analysis (*t*-test p-values) and predictive performance (AUC) for each lipid species. **(E–G)** ROC curves for the top predictive LPC species: **(E)** LPC(20:0), **(F)** LPC(20:2), and **(G)** LPC(18:0). PCA, principal component analysis; AUC, area under the curve, ROC, receiver operating characteristic; LPC, lysophosphatidylcholine.

To investigate the association between pre-treatment serum lipid profiles and the efficacy of nivolumab, we initially conducted a semi-targeted phospholipid analysis on serum samples from patients with NSCLC (**Figures 1**, **2A**). PCA of the detected phospholipid profiles revealed a clear separation between responder and non-responder groups (PERMANOVA p = 0.027; **Figure 2B**), indicating distinct lipidomic differences associated with treatment outcomes. Considering the results of the loading plot as well, this separation appeared to be primarily driven by variations in lysophosphatidylcholine (LPC) species (**Supplementary Figure 1A**). This finding was further supported by hierarchical clustering based on the most differentially abundant phospholipids, which demonstrated a clear classification of patients consistent with their clinical response status (**Figure 2C**). These analyses visually highlighted that several LPC species were elevated in the responder group compared to the non-responder group (**Supplementary Figure 1B**). To quantitatively assess the predictive potential of individual lipids, univariate analyses were conducted using Student’s t-tests, and the area under the receiver operating characteristic curve (AUC) was calculated for each lipid (**Figure 2D**). Among them, LPC(20:0) emerged as the most powerful predictor, exhibiting the highest discriminatory performance with an AUC of 0.782. Other LPC species, including LPC(20:2) and LPC(18:0), also showed significant predictive capability. To assess the risk of overfitting in this single-cohort study, we performed two independent internal validation analyses. Leave-One-Out Cross-Validation (LOOCV) using logistic regression demonstrated that LPC(20:0) maintained robust predictive performance with a cross-validated AUC of 0.720 (95% CI: 0.552–0.861), confirming that the discriminatory signal is not attributable to overfitting. Similarly, .632 Bootstrap Validation yielded an AUC of 0.781 for LPC(20:0), closely matching the standard AUC of 0.782 (**Figure 2E**). Consistent performance was observed across three candidate LPC species, with LOOCV AUCs ranging from 0.668 to 0.720 (**Figures 2F, G**; **Supplementary Figure 2**). These results confirm that the predictive signal of LPC species is robust under internal cross-validation.

### 3.2 Serum proteome profiling elucidates immune and metabolic modules associated with nivolumab response

**Figure 3:**
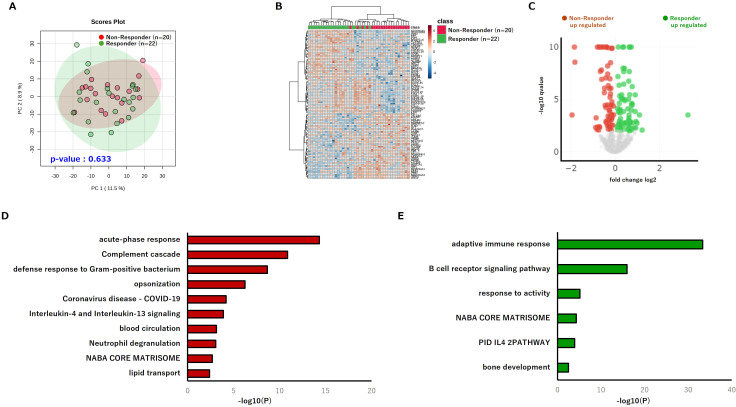
Serum proteome profiling reveals immune and metabolic modules associated with nivolumab response. **(A)** PCA plot of the pre-treatment serum proteome (n = 22 Responders; n = 20 Non-Responders) (PERMANOVA p = 0.633). **(B)** Hierarchical clustering heatmap based on the top 100 most variable proteins. **(C)** Volcano plot identifying proteins significantly differentially abundant between Responders and Non-Responders. **(D)** Functional enrichment analysis of proteins significantly upregulated in the Non-Responder group. **(E)** Functional enrichment analysis of proteins significantly upregulated in the Responder group. PCA, principal component analysis; PERMANOVA, permutational multivariate analysis of variance.

Quantitative serum proteomic profiling was performed using pre-treatment samples from the same patient cohort. Among more than 1,000 quantified proteins, in contrast to the phospholipid profiles, PCA revealed no significant overall separation between groups (PERMANOVA p = 0.633; **Figure 3A**). This suggests that analyses using all measured serum proteome data may not fully achieve patient stratification, suggesting that feature-level selection or differential abundance analysis is required to identify proteomic signatures relevant to treatment response. Therefore, hierarchical clustering of the top 100 variable proteins revealed partial segregation between Responder and Non-Responder samples (**Figure 3B**). Furthermore, volcano plot analysis identified proteins significantly enriched in either Non-Responder or Responder (**Figure 3C**). Functional enrichment analysis showed that Non-Responder enriched proteins were overrepresented in pathways related to “acute-phase response” and “complement and coagulation cascades” (**Figure 3D**), whereas Responder enriched proteins were significantly associated with “adaptive immune processes” and related pathways (**Figure 3E**). Collectively, these findings indicate that Non-Responders to PD-1 blockade exhibit elevated levels of inflammation-related factors even prior to treatment, whereas Responders display proteomic signatures suggestive of a primed adaptive immune system. These data reveal distinct immunological backgrounds between the two clinical groups.

### 3.3 Serum extracellular vesicle proteome profiling reveals distinct immune-related signatures associated with PD-1 response status

**Figure 4:**
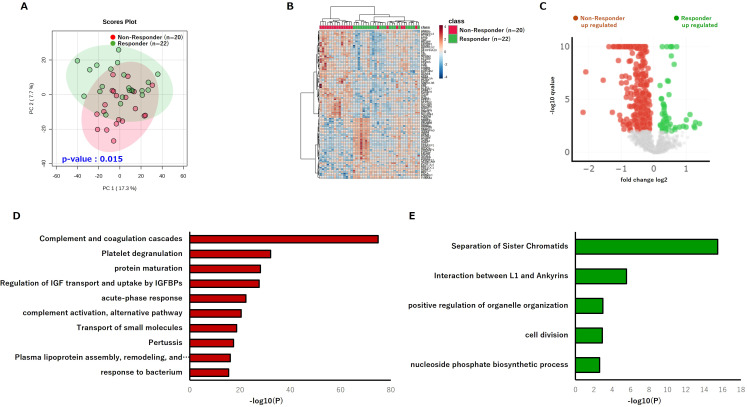
Serum EV proteome profiling reveals distinct immune-related signatures associated with PD-1 response status. **(A)** PCA plot of the pre-treatment serum EV proteome (n = 22 Responders; n = 20 Non-Responders) (PERMANOVA p = 0.015). **(B)** Hierarchical clustering heatmap based on the top 100 most variable EV proteins. **(C)** Volcano plot identifying EV proteins significantly differentially abundant between Responders and Non-Responders. **(D)** Functional enrichment analysis of EV proteins significantly upregulated in the Non-Responder group. **(E)** Functional enrichment analysis of EV proteins significantly upregulated in the Responder group. PCA, principal component analysis; PERMANOVA, permutational multivariate analysis of variance; EV, extracellular vesicle.

Following the analyses of phospholipids and serum proteins, we subsequently focused on another class of circulating mediators and performed quantitative proteomic profiling of serum-derived EV collected prior to nivolumab administration. First, to confirm that EV enrichment was achieved, we verified the presence of established EV markers in the proteomic data, including tetraspanins (CD9, CD63, CD81), flotillins (FLOT1, FLOT2), and ESCRT-associated proteins (TSG101, ALIX, VPS4A, VPS4B, CHMP4B). (**Supplementary Figure 3**). Among more than 3000 identified proteins, PCA revealed a significant separation between Responders and Non-Responders, indicating that the EV proteome captures biologically meaningful heterogeneity associated with therapeutic outcome ((PERMANOVA p = 0.015; **Figure 4A**). Hierarchical clustering of the top 100 most variable proteins demonstrated distinct grouping patterns between Responder and Non-Responder samples (**Figure 4B**). Moreover, volcano plot analysis identified subsets of proteins significantly enriched in each group (**Figure 4C**). Functional enrichment analysis revealed that proteins upregulated in the Non-Responder group were significantly overrepresented in pathways such as complement and coagulation cascades, platelet degranulation, and acute-phase response (**Figure 4D**). In contrast, proteins upregulated in the Responder group were enriched in pathways including separation of sister chromatids (**Figure 4E**). These results are consistent with the inflammatory and stress-related proteomic signatures observed in the serum of Non-Responders. Collectively, the serum EV proteome reflects the pre-treatment immune state of patients and suggests that vesicle-mediated intercellular communication may contribute to shaping a systemic immune environment conducive to nivolumab efficacy.

### 3.4 Lipid–proteome integration reveals LPC-linked networks bridging metabolism and immunity

**Figure 5:**
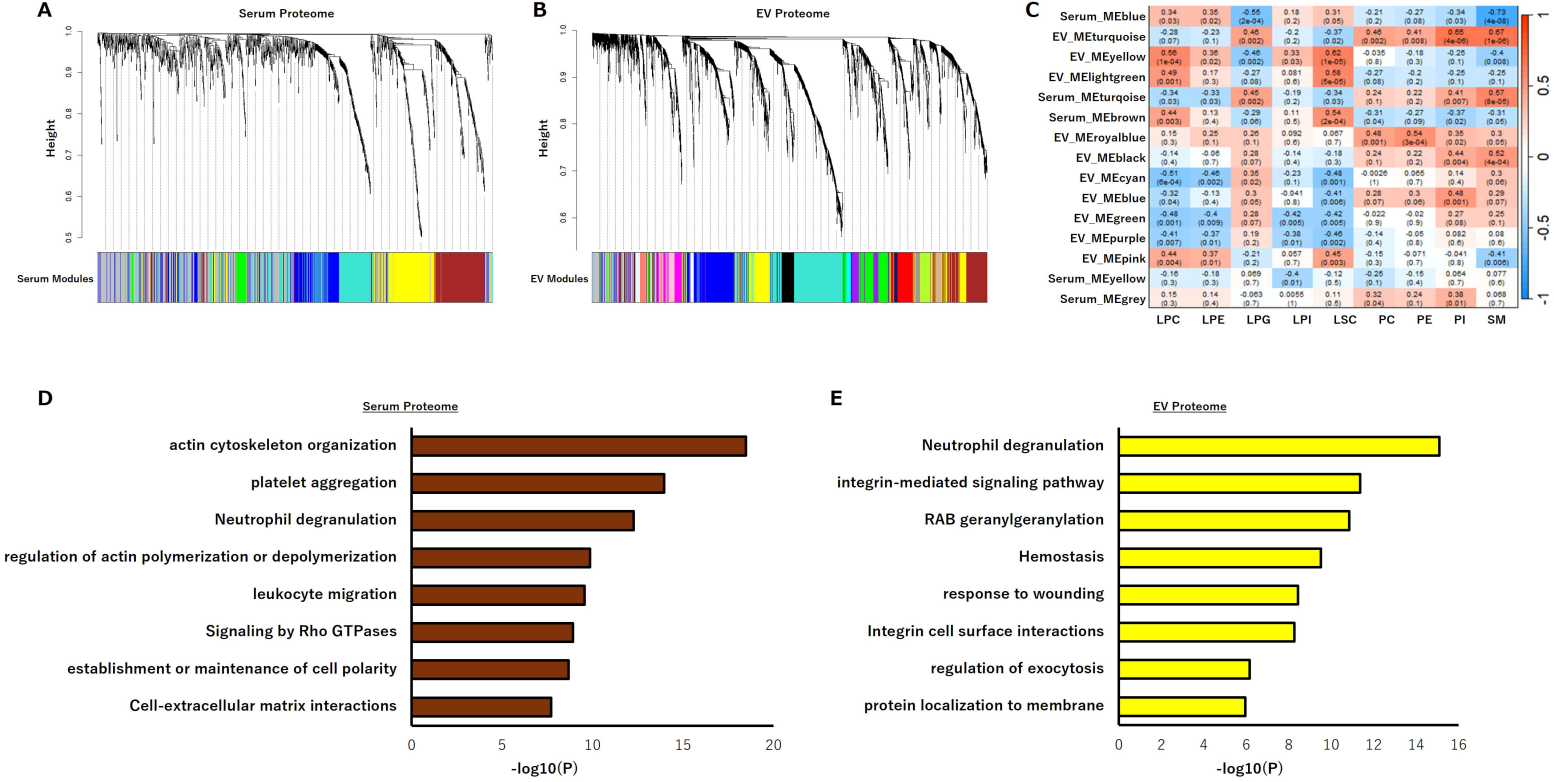
Lipid-proteome integration by WGCNA reveals LPC-associated networks. **(A, B)** Co-expression modules identified by WGCNA in the **(A)** serum proteome and **(B)** EV proteome. **(C)** Heatmap showing cross-modular correlations between proteome modules and phospholipid classes. **(D)** Enrichment analysis of the brown serum proteome module correlated with LPC. **(E)** Enrichment analysis of the yellow EV proteome module correlated with LPC. WGCNA, weighted gene co-expression network analysis; EV, extracellular vesicle, LPC, lysophosphatidylcholine.

To interpret the serum lipid signature within the context of systemic immunometabolic networks, we performed Weighted Gene Co-expression Network Analysis (WGCNA) integrating serum and EV proteomes with phospholipid classes. Multiple co-expression modules were identified within each proteome, forming biologically coherent clusters (**Figures 5A, B**). Cross-modular correlation analysis revealed modules significantly associated with phospholipid classes linked to nivolumab responsiveness (**Figure 5C**). In the serum proteome, the brown module showed the strongest positive correlation with LPC levels and was enriched for immune-related pathways such as “platelet aggregation” and “neutrophil degranulation” (**Figure 5D**). These findings suggest that the regulation of circulating LPC levels and immune homeostasis are co-regulated at the proteomic level. In the EV proteome, the yellow module also exhibited a significant positive correlation with LPC, with enrichment again observed for the “neutrophil degranulation” pathway (**Figure 5E**). Together, these results indicate that circulating LPC levels correlate with protein modules involved in platelet and neutrophil activation, reflecting a systemic immunometabolic state that may underlie responsiveness to PD-1 blockade therapy.

#### 3.4.1 Multilayer factor analysis identifies an LPC-linked latent axis integrating lipid metabolism with platelet and neutrophil activity

**Figure 6:**
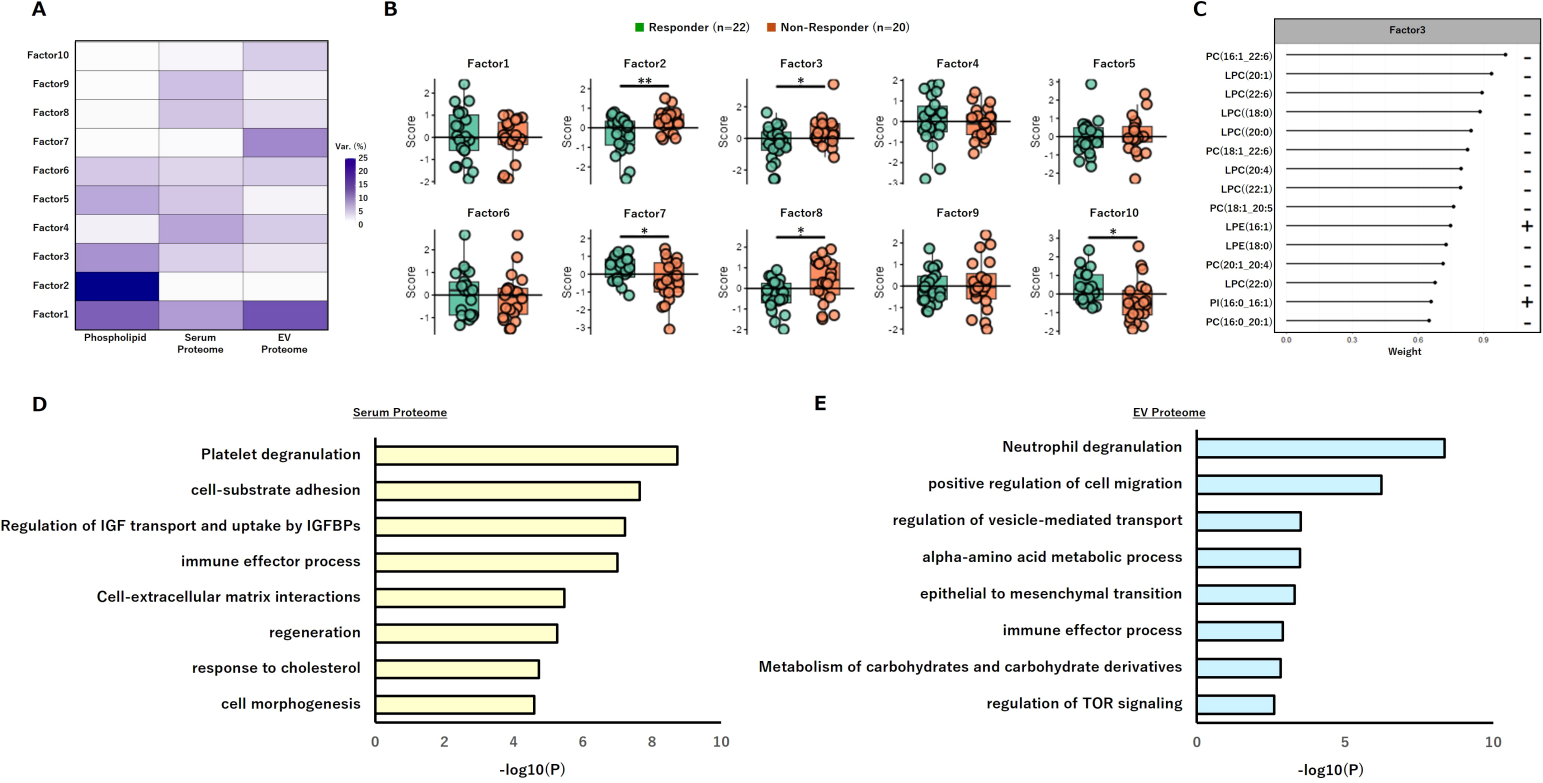
Multi-layer omics analysis by MOFA2 identifies an LPC-associated latent factor integrating lipid metabolism with platelet and neutrophil activity. **(A)** Schematic overview of the MOFA2 model integrating three layers: phospholipids, serum proteome, and EV proteome. **(B)** Score distribution of a latent factor showing significant differences between Responders and Non-Responders. Statistical significance was assessed using the Wilcoxon rank-sum test with Benjamini–Hochberg correction; asterisks indicate significance levels: *p < 0.05, **p < 0.01. **(C)** Loadings plot for the primary discriminative factor (Factor 3). **(D)** Functional enrichment analysis of serum proteome proteins with high loadings on Factor 3. **(E)** Functional enrichment analysis of EV proteome proteins with high loadings on Factor 3. EV, extracellular vesicle; LPC, lysophosphatidylcholine, MOFA, multi-omics factor analysis.

In addition to the correlation-based approach of WGCNA, we applied Multi-Omics Factor Analysis (MOFA) to extract shared variance structures across the phospholipid, serum-proteome, and EV-proteome layers (**Figure 6A**). Among the ten inferred latent factors, several showed significant differences between responders and non-responders (**Figure 6B**). Notably, Factor 3, one of the discriminative components, received the strongest contribution from LPC species—specifically LPC(20:0), LPC(20:1), LPC(22:0), and LPC(22:6)—all of which exhibited negative loadings (**Figures 6C**; **Supplementary Figure 4**). These results indicate that this latent component is primarily associated with LPC. To further characterize the biological signatures associated with Factor 3, we performed functional enrichment analysis of proteins with high loadings. In the serum proteome, pathways related to platelet activation were significantly overrepresented (**Figure 6D**), whereas in the EV proteome, proteins involved in neutrophil activation were highly enriched (**Figure 6E**). These trends were consistent with the co-expression modules identified by WGCNA. Collectively, the LPC-enriched latent axis appears to function as an integrative network linking lipid metabolism with platelet and neutrophil activity, reflecting a systemic immunometabolic state that may underlie responsiveness to PD-1 blockade therapy.

## 4 Discussion

This study elucidated a significant association between the host’s systemic immunometabolic state and the therapeutic response to PD-1 blockade in NSCLC. By integrating multi-omics data from pre-treatment serum samples, we identified LPC as a potential biomarker predictive of nivolumab efficacy. A distinctive feature of this study is that the LPC signature appears to not only serve as a promising predictive biomarker of therapeutic response, but also reflects pre-existing immune-related proteomic activity—an indicator of the host’s immunometabolic state before therapy. These findings support the concept that the metabolic context, as reflected by circulating lipids, may be associated with or reflect differences in immune competence.

We aimed to develop liquid biopsy biomarkers, and performed omics analyses focused on circulating phospholipids using pre-treatment serum samples from patients with NSCLC who were treated with nivolumab. Consequently, we identified the LPC class as a potential marker for patient stratification. Our findings are supported by the report by Iwasaki et al. This report demonstrated that plasma LPC levels function as a reliable candidate biomarker in a cohort of 149 patients with esophageal squamous cell carcinoma or head and neck squamous cell carcinoma (23). Iwasaki et al. reported that plasma LPC levels correlated with systemic inflammatory status (Glasgow Prognosis Score) in patients with esophageal squamous cell carcinoma or head and neck squamous cell carcinoma, and that 10 types of LPC showed a significant negative correlation with prognosis. Subgroup analysis suggested an association between elevated total LPC levels and improved OS in the primary ICI treatment group. However, prediction was based on total LPC values dichotomized at the median; AUC analysis at the species level was not performed, and the endpoint was OS rather than objective ICI response. Integrated with our current findings limited to pre-treatment specimens in NSCLC, these results suggest that LPCs may extend their potential as biomarkers for ICI response across different cancer types. The biological importance of LPC as a predictive signature lies in its dual nature—as both a metabolic intermediate and an immune signaling molecule. LPC is generated by phospholipase A_2_-mediated cleavage of phosphatidylcholine and circulates bound to albumin or lipoproteins (24). It exerts pleiotropic effects on multiple immune cells, including T cells, and macrophages (25, 26). Notably, the LPC transporter MFSD2A is upregulated on activated CD8^+^ T cells and has been shown to be essential for memory T cell maintenance, suggesting that adequate LPC availability directly supports sustained CD8^+^ T cell-mediated immunity (27). Therefore, the “high-LPC state” observed in responders may be associated with differences in immune-cell migration, activation thresholds, and effector functions. Supporting this, a recent study demonstrated that circulating LPC(18:2) levels decline prior to severe immune-related adverse events following immune checkpoint blockade, and that LPC(18:2) supplementation suppressed neutrophilia without impairing anti-tumor immunity, directly linking LPC homeostasis to immune regulation in the ICI setting (28).

A key strength of our research lies in exploring the host’s biological background linked to lipid concentration changes of uncertain immunological interpretation by integrating serum and EV proteomic analyses, rather than by evaluating lipids as biomarkers in isolation. When comparing nivolumab Responders and Non-Responders, we observed an increase in acute-phase and complement-related proteins in the non-responder group. This finding is consistent with previous reports demonstrating that elevated levels of acute-phase proteins, such as C-reactive protein, in pre-treatment serum are associated with poor nivolumab efficacy (29, 30). Moreover, combinatorial therapeutic approaches that inhibit both complement activation and immune-checkpoint signaling have been investigated in other studies (30). Therefore, our patient cohort is unlikely to deviate from established clinical contexts.

One of the notable findings of this study is that both WGCNA and MOFA2 analyses linked the LPC signature to proteomic pathways involved in platelet and neutrophil biology. Given that an elevated neutrophil-to-lymphocyte ratio (NLR) is a known predictor of poor prognosis in ICI therapy (31), it is crucial to examine the potential regulation of neutrophil function by LPC. Neutrophil extracellular traps (NETs) have garnered attention as factors that suppress anti-tumor immunity and limit ICI efficacy (32, 33). Interestingly, LPC has been reported to inhibit NET formation, raising the possibility that LPC may be associated with a more immunocompetent systemic state. Taken together with our correlative observations, these findings are consistent with a relatively immunostimulatory host environment, though causal relationships remain to be established (34, 35). On the other hand, an elevated platelet-to-lymphocyte ratio (PLR) is also an indicator of poor prognosis (36). This clinical observation aligns with our analytical results, which demonstrated that proteins related to platelet degranulation were abundant in serum EVs from Non-Responders group. The impact of LPC on platelets appears to be dual-faceted: while it promotes activation, aggregation, and vascular inflammation (37), it has also been reported to induce oxidative stress and platelet cell death (38). These opposing effects suggest a dependency on the microenvironmental conditions, and the net impact of LPC–platelet interactions during ICI therapy remains unclear.

Importantly, the integrated analyses in this study do not demonstrate causality. Platelet activation can induce LPC production via secretory phospholipase A2 (39). Furthermore, tumors responsive to ICI are often known to be immunologically “hot” (40); thus, immune activation within the tumor may drive cytokine production and subsequent systemic platelet activation, resulting in a secondary elevation of circulating LPC levels. Given these perspectives, whether ICI non-response and the regulation of circulating LPC levels stem from a unified platelet activity or are driven by distinct pathways has rarely been discussed. To gain further insight, we examined the overlap between the platelet degranulation-related proteins significantly enriched in EVs from the non-responder group and the platelet-related proteins associated with LPC identified by WGCNA (aggregation-related) and MOFA2 (degranulation-related) analyses. We found minimal overlap between these groups (**Supplementary Figure 5**). Enrichment analysis indicated that platelet activity was one of the dominant features in both analyses; however, minimal overlap may reflect multiple non-exclusive factors. These include methodological differences between analytical frameworks (DEA captures differential abundance, WGCNA captures LPC-correlated co-expression, MOFA2 captures latent factor loadings), limitations in statistical power due to small sample sizes, functional redundancy within platelet pathways, and potentially genuine biological divergence between platelet subsystems associated with ICI non-responsiveness and those linked to LPC regulation. Current correlative data cannot distinguish between these possibilities, and this question warrants investigation in larger independent cohorts or more direct experimental and biochemical approaches. The observed elevation in LPC likely reflects the activation of innate immunity or a related systemic immunometabolic state. Although direct causality requires further verification, the biological plausibility of these associations supports the role of LPC not merely as a surrogate for cell counts, but as a correlative indicator of a systemic immunometabolic state associated with treatment response.

Such circulating metabolic biomarkers provide a complementary perspective to tumor-intrinsic predictors such as PD-L1 expression or tumor mutational burden. While tumor characteristics remain indispensable, they alone cannot fully explain the host’s capacity to mount an effective immune response. For instance, the gut microbiota’s influence on ICI responsiveness exemplifies how immune regulation in non-tumor tissues can affect therapeutic efficacy (41, 42). In this broader context, the LPC signature may function as a non-invasive indicator reflecting systemic immune potential—essentially serving as a mirror of overall immune tone. Incorporating this metabolic marker into clinical stratification frameworks could help identify patients who may still benefit from ICI therapy even when PD-L1 expression is low.

Multiple metabolomics and lipidomics studies have investigated circulating biomarkers of ICI response in NSCLC, but their findings differ from ours both in terms of analytical methods and the identified metabolite classes. Ghini et al. applied serum NMR-based metabolomics to NSCLC patients receiving nivolumab or pembrolizumab, identifying a signature centered on alanine and pyruvate (AUC = 0.79) (43). Lee et al. combined targeted metabolic profiling with machine learning in a large NSCLC cohort, identifying amino acid ratios (relative values of histidine and homocysteine, phenylalanine, and sarcosine) as strong predictors of ICI efficacy and suggesting glycolytic metabolites like lactate as outcome determinants (44). Jiang et al. applied untargeted plasma lipidomics to NSCLC patients receiving chemo-immunotherapy and identified phosphatidylcholine species as the most discriminative lipids (AUC = 0.85) (45). While PC and LPC share a choline headgroup structure, LPC species were not identified as predictors, which may reflect differences in treatment regimens, sample types, or distinct immunomodulatory properties between intact PC and its hydrolysis products. Xu et al. analyzed plasma metabolomes at the pathway level from two independent NSCLC cohorts receiving anti-PD-1 therapy, identifying bile acid biosynthesis and choline metabolism as the most significantly enriched pathways associated with survival (46). Taken together, these studies demonstrate that ICI responsiveness in NSCLC involves reprogramming of circulating metabolism across multiple metabolite classes. This study complements and extends these findings by establishing LPC species within an integrated multi-omics framework, proposing a mechanistic rationale for LPC species as liquid biopsy biomarker candidates in NSCLC.

From a translational perspective, this study offers two promising directions. First, serum LPC is an easily accessible biomarker measurable by standard mass-spectrometry platforms, supporting prospective validation and clinical implementation (47). Second, the LPC axis itself may serve as a therapeutic target. Considering its potential immunomodulatory effects, regulating systemic lipid metabolism through dietary intervention or pharmacological analogues of LPC could modulate the host’s systemic metabolic environment in a manner that enhances immunotherapy responsiveness. This pathway warrants further experimental validation (48).

However, the limitations of this study should be acknowledged. The analysis was conducted using a single dataset with a retrospective study design and a relatively small cohort size. Although cross-validation was performed within the cohort, the absence of an independent external validation cohort remains a significant challenge. Prospective validation in an independent NSCLC cohort receiving nivolumab is therefore an essential next step before clinical translation. The present study should be considered a hypothesis-generating analysis. Furthermore, all patients in this cohort had adenocarcinoma histology. While this excludes histological heterogeneity as a confounding factor, it also limits direct generalization to squamous cell carcinoma or other NSCLC subtypes. Reflecting clinical practice at the time of enrollment, many specimens lacked PD-L1 expression data. This further underscores the need for prospective validation using new specimens. The semi-targeted lipidomic platform used in this study is optimized for phospholipid profiling and does not capture other bioactive lipid classes known as immunomodulators, such as sphingolipids, ceramides, and eicosanoids. Given that each lipid class is also suspected to be associated with immunity or cancer, future studies should adopt a non-targeted platform. Regarding the EV characterization analyzed in this study, while the identification of multiple proteins involved in EV quality control suggested successful EV concentration, formal MISEV2023 characterization, including nanoparticle tracking analysis and electron microscopy, was not performed. In this respect, the results should be recognized as based on proteomics-based QC evidence obtained through Tim4-mediated phosphatidylserine-affinity EV isolation, rather than formal particle characterization. Our multi-omics analysis showed consistent correlations but did not establish causality. Whether LPCs directly enhance immune function or merely serve as surrogate markers of favorable immune status remains a question to be clarified. Furthermore, confounding factors affecting the lipid profile, such as diet, comorbidities, and gut microbiota composition, must be strictly controlled in future studies. Functional analysis is required to elucidate the molecular pathways by which LPC modulates platelet, neutrophil, and lymphocyte activity in the context of cancer immunity. Establishing the mechanistic validity of the observed correlations necessitates *in vitro* functional validation of LPC’s effects on immune cell activation and antitumor immunity.In conclusion, this study bridges systemic metabolism and tumor immunology by demonstrating that the serum LPC signature is a candidate predictive biomarker of response to immune-checkpoint blockade. These findings suggest that the host’s immunometabolic tone may be a key determinant of therapeutic efficacy and underscore the utility of a multi-omics analytical approach. The LPC-centered network linking lipid metabolism with innate-immune activation provides a biological rationale for developing new biomarkers and therapeutic strategies, ultimately contributing to the advancement of personalized medicine in NSCLC.

## Data Availability

The datasets presented in this study can be found in online repositories. The names of the repository/repositories and accession number(s) can be found in the article/**Supplementary Material**.

## Supplementary material

Supplementary Figure 1 **(A)** Biplot of the PCA of serum phospholipids. **(B)** Bar graphs comparing the levels of LPC species identified from serum phospholipid analysis between Responders and Non-Responders, determined by Student’s *t*-test. Error bars represent standard deviation. p < 0.05 (*), p < 0.01 (**). LPC, lysophosphatidylcholine; PCA, principal component analysis.

Supplementary Figure 2 Standard AUC values were calculated using all samples (n = 42), with sensitivity and specificity determined at the Youden’s J optimal cutoff. Ninety-five percent confidence intervals were estimated by bootstrap resampling (B = 2,000 iterations). Cross-validation performance was assessed by leave-one-out cross-validation (LOOCV), with 95% CI estimated by bootstrap resampling of LOOCV predictions. The.632 bootstrap AUC was calculated as a bias-corrected estimate of predictive performance. AUC, area under the receiver operating characteristic curve; CI, confidence interval; EV, extracellular vesicle; LPC, lysophosphatidylcholine; LOOCV, leave-one-out cross-validation; NPV, negative predictive value; PPV, positive predictive value; Sens, sensitivity; Spec, specificity.

Supplementary Figure 3 EV proteome quality assessment based on protein marker detection. Scatter plot comparing mean log_2_ protein intensity between the serum proteome and EV proteome across all samples (n = 42). Gray points represent all proteins detected in both fractions. Known EV-associated markers are highlighted: tetraspanins (CD9, CD63, CD81, CD151; blue), ESCRT/biogenesis-associated proteins (TSG101, ALIX/PDCD6IP, VPS4A, VPS4B, CHMP4B; green), flotillins (FLOT1, FLOT2; orange), and co-chaperones (HSPA8, HSP90AA1, SDCBP; purple). Proteins detected exclusively in the EV fraction (EV-exclusive) are indicated along the left shaded region. Serum-abundant proteins (ALB, A2M, FGA) serve as reference controls. Dashed lines indicate the identity line (no difference) and +3/+6 log_2_ enrichment thresholds. ALB, albumin; ESCRT, endosomal sorting complexes required for transport; EV, extracellular vesicle.

Supplementary Figure 4 Loading plots of MOFA2 factors 2, 7, 8, and 10, which showed significant differences between Responders and Non-Responders. MOFA, multi-omics factor analysis.

Supplementary Figure 5 Venn diagram depicting the intersection of three protein sets: (1) Platelet degranulation proteins enriched in EVs from Non-Responders, (2) LPC-correlated platelet aggregation-related proteins (WGCNA), and (3) LPC-associated platelet degranulation-related proteins (MOFA2).

Supplementary Table 1 Results of serum phospholipid profiling.

Supplementary Table 2 Results of serum proteome analysis.

Supplementary Table 3 Results of serum EV proteome analysis.

Supplementary Table 4 Enrichment analysis of proteins upregulated in non-responders based on serum proteome analysis.

Supplementary Table 5 Enrichment analysis of proteins upregulated in responders based on serum proteome analysis.

Supplementary Table 6 Enrichment analysis of proteins upregulated in non-responders based on serum EV proteome analysis.

Supplementary Table 7 Enrichment analysis of proteins upregulated in responders based on serum EV proteome analysis.

Supplementary Table 8 Enrichment analysis of serum proteins correlated with serum LPC classes based on WGCNA.

Supplementary Table 9 Enrichment analysis of serum EV proteins correlated with serum LPC classes based on WGCNA.

Supplementary Table 10 Enrichment analysis of serum proteins with high loadings for Factor 3 based on MOFA2 analysis.

Supplementary Table 11 Enrichment analysis of serum EV proteins with high loadings for Factor 3 based on MOFA2 analysis.

## References

[1] Pirker R . Immunotherapy combinations in advanced nonsmall cell lung cancer. Curr Opin Oncol. (2021) 33:73–9. doi: 10.1097/CCO.0000000000000700. PMID: 33186185

[2] Tang S Qin C Hu H Liu T He Y Guo H . Immune checkpoint inhibitors in non-small cell lung cancer: Progress, challenges, and prospects. Cells. (2022) 11:320. doi: 10.3390/cells11030320. PMID: 35159131 PMC8834198

[3] Mountzios G Remon J Hendriks LEL García-Campelo R Rolfo C Van Schil P . Immune-checkpoint inhibition for resectable non-small-cell lung cancer-opportunities and challenges. Nat Rev Clin Oncol. (2023) 20:664–77. doi: 10.1038/s41571-023-00794-7. PMID: 37488229

[4] Nagasaki J Ishino T Togashi Y . Mechanisms of resistance to immune checkpoint inhibitors. Cancer Sci. (2022) 113:3303–12. doi: 10.1111/cas.15497. PMID: 35848888 PMC9530865

[5] Bagchi S Yuan R Engleman EG . Immune checkpoint inhibitors for the treatment of cancer: Clinical impact and mechanisms of response and resistance. Annu Rev Pathol. (2021) 16:223–49. doi: 10.1146/annurev-pathol-042020-042741. PMID: 33197221

[6] Karasarides M Cogdill AP Robbins PB Bowden M Burton EM Butterfield LH . Hallmarks of resistance to immune-checkpoint inhibitors. Cancer Immunol Res. (2022) 10:372–83. doi: 10.1158/2326-6066.CIR-20-0586, PMID: 35362046 PMC9381103

[7] Cook S Samuel V Meyers DE Stukalin I Litt I Sangha R . Immune-related adverse events and survival among patients with metastatic NSCLC treated with immune checkpoint inhibitors. JAMA Netw Open. (2024) 7:e2352302. doi: 10.1001/jamanetworkopen.2023.52302. PMID: 38236598 PMC10797458

[8] Verheijden RJ de Groot JS Fabriek BO Hew MN May AM Suijkerbuijk KPM . Corticosteroids for immune-related adverse events and checkpoint inhibitor efficacy: Analysis of six clinical trials. J Clin Oncol. (2024) 42:3713–24. doi: 10.1200/JCO.24.00191. PMID: 39110922

[9] Şen GA Öztaş NŞ Değerli E Safarov S Guliyev M Bedir Ş . Effects of immune related adverse events and corticosteroids on the outcome of patients treated with immune checkpoint inhibitors. Sci Rep. (2025) 15:6310. doi: 10.1038/s41598-025-91102-z. PMID: 39984593 PMC11845686

[10] Bodor JN Boumber Y Borghaei H . Biomarkers for immune checkpoint inhibition in non-small cell lung cancer (NSCLC). Cancer. (2020) 126:260–70. doi: 10.1002/cncr.32468. PMID: 31691957 PMC7372560

[11] Yamaguch H Hsu JM Sun L Wang SC Hung MC . Advances and prospects of biomarkers for immune checkpoint inhibitors. Cell Rep Med. (2024) 5:101621. doi: 10.1016/j.xcrm.2024.101621. PMID: 38906149 PMC11293349

[12] Vokes NI Liu D Ricciuti B Jimenez-Aguilar E Rizvi H Dietlein F . Harmonization of tumor mutational burden quantification and association with response to immune checkpoint blockade in non-small-cell lung cancer. JCO Precis Oncol. (2019) 3:PO.19.00171. doi: 10.1200/PO.19.00171. PMID: 31832578 PMC6907021

[13] Ricciuti B Wang X Alessi JV Rizvi H Mahadevan NR Li YY . Association of high tumor mutation burden in non-small cell lung cancers with increased immune infiltration and improved clinical outcomes of PD-L1 blockade across PD-L1 expression levels. JAMA Oncol. (2022) 8:1160–8. doi: 10.1001/jamaoncol.2022.1981. PMID: 35708671 PMC9204620

[14] Spagnolo CC Pepe F Ciappina G Nucera F Ruggeri P Squeri A . Circulating biomarkers as predictors of response to immune checkpoint inhibitors in NSCLC: Are we on the right path? Crit Rev Oncol Hematol. (2024) 197:104332. doi: 10.1016/j.critrevonc.2024.104332. PMID: 38580184

[15] Ma L Guo H Zhao Y Liu Z Wang C Bu J . Liquid biopsy in cancer current: status, challenges and future prospects. Signal Transduct Targeted Ther. (2024) 9:336. doi: 10.1038/s41392-024-02021-w. PMID: 39617822 PMC11609310

[16] Ping Y Fan Q Zhang Y . Modulating lipid metabolism improves tumor immunotherapy. J Immunother Cancer. (2025) 13:e010824. doi: 10.1136/jitc-2024-010824. PMID: 39904563 PMC11795363

[17] Choi JH Kagan JC . Oxidized phospholipid damage signals as modulators of immunity. Open Biol. (2025) 15:240391. doi: 10.1098/rsob.240391. PMID: 40730233 PMC12307067

[18] Delmas D Mialhe A Cotte AK Connat JL Bouyer F Hermetet F . Lipid metabolism in cancer: Exploring phospholipids as potential biomarkers. BioMed Pharmacother. (2025) 187:118095. doi: 10.1016/j.biopha.2025.118095. PMID: 40311223

[19] Pang Z Lu Y Zhou G Hui F Xu L Viau C . MetaboAnalyst 6.0: towards a unified platform for metabolomics data processing, analysis and interpretation. Nucleic Acids Res. (2024) 52:W398–406. doi: 10.1093/nar/gkae253. PMID: 38587201 PMC11223798

[20] Tyanova S Temu T Sinitcyn P Carlson A Hein MY Geiger T . The Perseus computational platform for comprehensive analysis of (prote)omics data. Nat Methods. (2016) 13:731–40. doi: 10.1038/nmeth.3901. PMID: 27348712

[21] Koopmans F Li KW Klaassen RV Smit AB . MS-DAP platform for downstream data analysis of label-free proteomics uncovers optimal workflows in benchmark data sets and increased sensitivity in analysis of Alzheimer's biomarker data. J Proteome Res. (2023) 22:374–86. doi: 10.1021/acs.jproteome.2c00513. PMID: 36541440 PMC9903323

[22] Zhou Y Zhou B Pache L Chang M Khodabakhshi AH Tanaseichuk O . Metascape provides a biologist-oriented resource for the analysis of systems-level datasets. Nat Commun. (2019) 10:1523. doi: 10.1038/s41467-019-09234-6. PMID: 30944313 PMC6447622

[23] Iwasaki T Shirota H Hishinuma E Kawaoka S Matsukawa N Kasahara Y . Plasma lysophosphatidylcholine levels correlate with prognosis and immunotherapy response in squamous cell carcinoma. Int J Mol Sci. (2025) 26:7528. doi: 10.3390/ijms26157528. PMID: 40806656 PMC12347404

[24] Law SH Chan ML Marathe GK Parveen F Chen CH Ke LY . An updated review of lysophosphatidylcholine metabolism in human diseases. Int J Mol Sci. (2019) 20:1149. doi: 10.3390/ijms20051149. PMID: 30845751 PMC6429061

[25] Liu X Hartman CL Li L Albert CJ Si F Gao A . Reprogramming lipid metabolism prevents effector T cell senescence and enhances tumor immunotherapy. Sci Transl Med. (2021) 13:eaaz6314. doi: 10.1126/scitranslmed.aaz6314. PMID: 33790024 PMC12040281

[26] Oya Y Yoshida T Kuroda H Mikubo M Kondo C Shimizu J . Predictive clinical parameters for the response of nivolumab in pretreated advanced non-small-cell lung cancer. Oncotarget. (2017) 8:103117–28. doi: 10.18632/oncotarget.21602. PMID: 29262550 PMC5732716

[27] Piccirillo AR Hyzny EJ Beppu LY Menk AV Wallace CT Hawse WF . The lysophosphatidylcholine transporter MFSD2A is essential for CD8+ memory T cell maintenance and secondary response to infection. Int J Mol Sci. (2019) 26:7528. doi: 10.3390/ijms26157528. PMID: 31127034 PMC6581627

[28] Mathews IT Saminathan P Henglin M Liu M Nadig N Fang C . Linoleoyl-lysophosphatidylcholine suppresses immune-related adverse events due to immune checkpoint blockade. medRxiv. (2024), 2024.08.07.24310974. doi: 10.1101/2024.08.07.24310974. PMID: 39148854 PMC11326322

[29] Schneider MA Rozy A Wrenger S Christopoulos P Muley T Thomas M . Acute phase proteins as early predictors for immunotherapy response in advanced NSCLC: An explorative study. Front Oncol. (2022) 12:772076. doi: 10.3389/fonc.2022.772076. PMID: 35174082 PMC8841510

[30] Ajona D Ortiz-Espinosa S Moreno H Lozano T Pajares MJ Agorreta J . A combined PD-1/C5a blockade synergistically protects against lung cancer growth and metastasis. Cancer Discov. (2017) 7:694–703. doi: 10.1158/2159-8290.CD-16-1184. PMID: 28288993

[31] Mosca M Nigro MC Pagani R De Giglio A Di Federico A . Neutrophil-to-lymphocyte ratio (NLR) in NSCLC, gastrointestinal, and other solid tumors: Immunotherapy and beyond. Biomolecules. (2023) 13:1803. doi: 10.3390/biom13121803. PMID: 38136673 PMC10741961

[32] Horaguchi S Nakahara Y Igarashi Y Kouro T Wei F Murotani K . Prognostic significance of plasma neutrophil extracellular trap levels in patients with non-small cell lung cancer treated with immune checkpoint inhibitors. Biomedicines. (2024) 12:1831. doi: 10.3390/biomedicines12081831. PMID: 39200295 PMC11351864

[33] Miao S Rodriguez BL Gibbons DL . The multifaceted role of neutrophils in NSCLC in the era of immune checkpoint inhibitors. Cancers (Basel). (2024) 16:2507. doi: 10.3390/cancers16142507. PMID: 39061147 PMC11274601

[34] Jeong S Kim B Byun DJ Jin S Seo BS Shin MH . Lysophosphatidylcholine alleviates acute lung injury by regulating neutrophil motility and neutrophil extracellular trap formation. Front Cell Dev Biol. (2022) 10:941914. doi: 10.3389/fcell.2022.941914. PMID: 35859904 PMC9289271

[35] Lin P Welch EJ Gao XP Malik AB Ye RD . Lysophosphatidylcholine modulates neutrophil oxidant production through elevation of cyclic AMP. J Immunol. (2005) 174:2981–9. doi: 10.4049/jimmunol.174.5.2981. PMID: 15728511

[36] Wang H Li C Yang R Jin J Liu D Li W . Prognostic value of the platelet-to-lymphocyte ratio in lung cancer patients receiving immunotherapy: A systematic review and meta-analysis. PloS One. (2022) 17:e0268288. doi: 10.1371/journal.pone.0268288. PMID: 35522679 PMC9075650

[37] Diehl P Nienaber F Zaldivia MTK Stamm J Siegel PM Mellett NA . Lysophosphatidylcholine is a major component of platelet microvesicles promoting platelet activation and reporting atherosclerotic plaque instability. Thromb Haemost. (2019) 119:1295–310. doi: 10.1055/s-0039-1683409. PMID: 31378855

[38] Yadav P Beura SK Panigrahi AR Kulkarni PP Yadav MK Munshi A . Lysophosphatidylcholine induces oxidative stress and calcium-mediated cell death in human blood platelets. Cell Biol Int. (2024) 48:1266–84. doi: 10.1002/cbin.12192. PMID: 38837523

[39] Blache D Gautier T Tietge UJ Lagrost L . Activated platelets contribute to oxidized low-density lipoproteins and dysfunctional high-density lipoproteins through a phospholipase A2-dependent mechanism. FASEB J. (2012) 26:927–37. doi: 10.1096/fj.11-191593. PMID: 22042222

[40] Havel JJ Chowell D Chan TA . The evolving landscape of biomarkers for checkpoint inhibitor immunotherapy. Nat Rev Cancer. (2019) 19:133–50. doi: 10.1038/s41568-019-0116-x. PMID: 30755690 PMC6705396

[41] Gopalakrishnan V Spencer CN Nezi L Reuben A Andrews MC Karpinets TV . Gut microbiome modulates response to anti-PD-1 immunotherapy in melanoma patients. Science. (2018) 359:97–103. doi: 10.1126/science.aan4236. PMID: 29097493 PMC5827966

[42] Ansaldo E Belkaid Y . How microbiota improve immunotherapy. Science. (2021) 373:966–7. doi: 10.1126/science.abl3656. PMID: 34446595

[43] Ghini V Laera L Fantechi B Monte FD Benelli M McCartney A . Metabolomics to assess response to immune checkpoint inhibitors in patients with non-small-cell lung cancer. Cancers. (2020) 12:3574. doi: 10.3390/cancers12123574. PMID: 33265926 PMC7760033

[44] Lee SH Kim S Lee J Kim Y Joo Y Heo JY . Comprehensive metabolomic analysis identifies key biomarkers and modulators of immunotherapy response in NSCLC patients. Drug Resist Update. (2024) 77:101159. doi: 10.1016/j.drup.2024.101159. PMID: 39405736

[45] Jiang H Li XS Yang Y Qi RX . Plasma lipidomics profiling in predicting the chemo-immunotherapy response in advanced non-small cell lung cancer. Front Oncol. (2024) 14:1348164. doi: 10.3389/fonc.2024.1348164. PMID: 39040440 PMC11260645

[46] Xu Y Ding K Peng Z Ding L Li H Fan Y . Evaluating for correlations between specific metabolites in patients receiving first-line or second-line immunotherapy for metastatic or recurrent NSCLC: An exploratory study based on two cohorts. Mol Cancer Ther. (2024) 23:733–42. doi: 10.1158/1535-7163.MCT-23-0459. PMID: 38346938 PMC11063768

[47] Engel KM Schiller J Galuska CE Fuchs B . Phospholipases and reactive oxygen species derived lipid biomarkers in healthy and diseased humans and animals-a focus on lysophosphatidylcholine. Front Physiol. (2021) 12:732319. doi: 10.3389/fphys.2021.732319 34858200 PMC8631503

[48] Maher TM Ford P Brown KK Costabel U Cottin V Danoff SK . Ziritaxestat, a novel autotaxin inhibitor, and lung function in idiopathic pulmonary fibrosis: The ISABELA 1 and 2 randomized clinical trials. JAMA. (2023) 329:1567–78. doi: 10.1001/jama.2023.5355. PMID: 37159034 PMC10170340

