# Supplementary figures and images for: Identification of predictive biomarkers for nivolumab efficacy in non-small cell lung cancer through integrated serum lipidomics and proteomics analysis
Source: Front Immunol. 2026 Apr 14;17:1773700. doi: 10.3389/fimmu.2026.1773700 (PMC13121081; doi:10.3389/fimmu.2026.1773700)

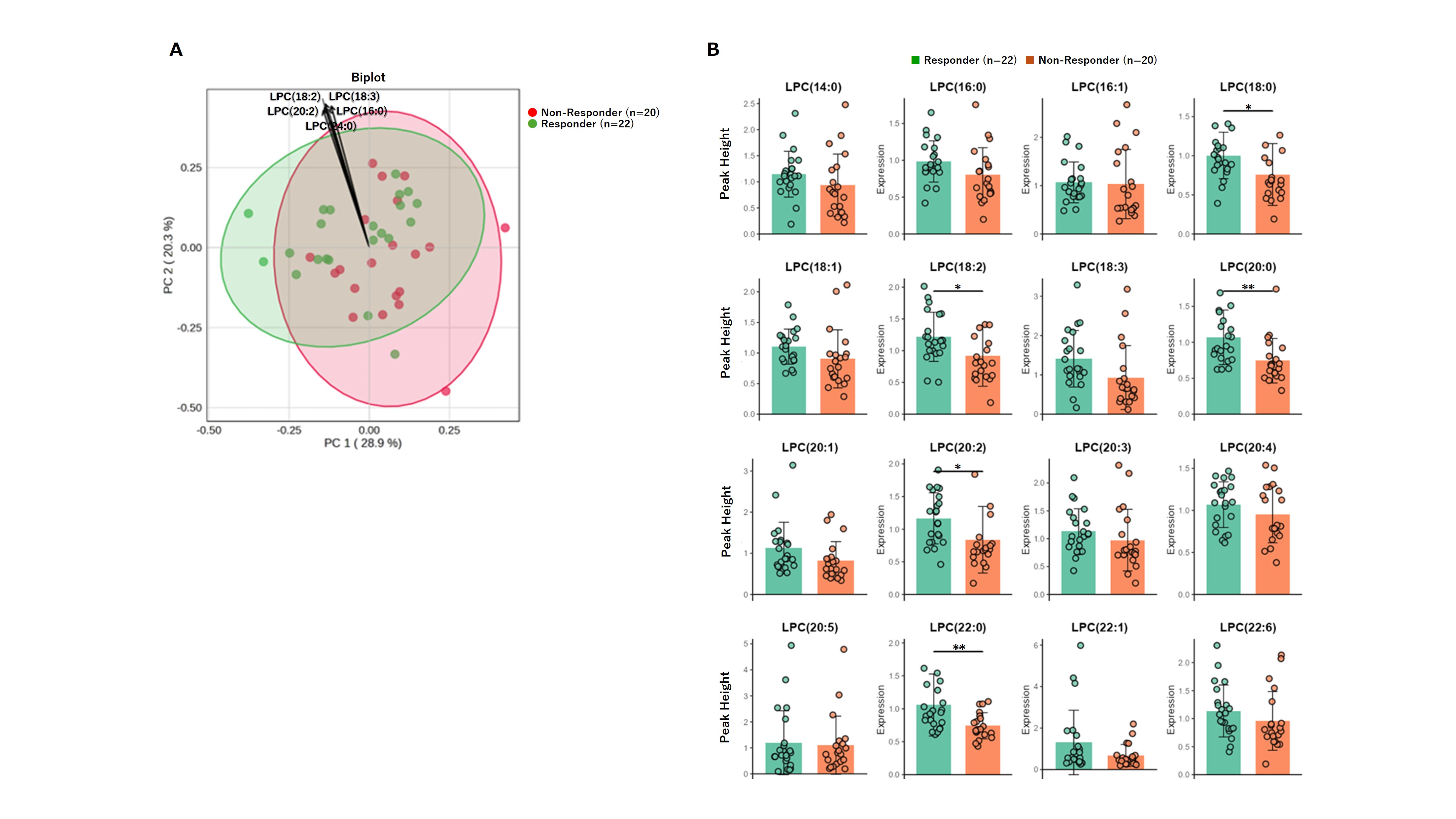

Supplement: Supplementary Figure 1 — (A) Biplot of the PCA of serum phospholipids. (B) Bar graphs comparing the levels of LPC species identified from serum phospholipid analysis between Responders and Non-Responders, determined by Student’s t-test. Error bars represent standard deviation. p < 0.05 (*), p < 0.01 (**). LPC, lysophosphatidylcholine; PCA, principal component analysis. [file Image1.jpeg]

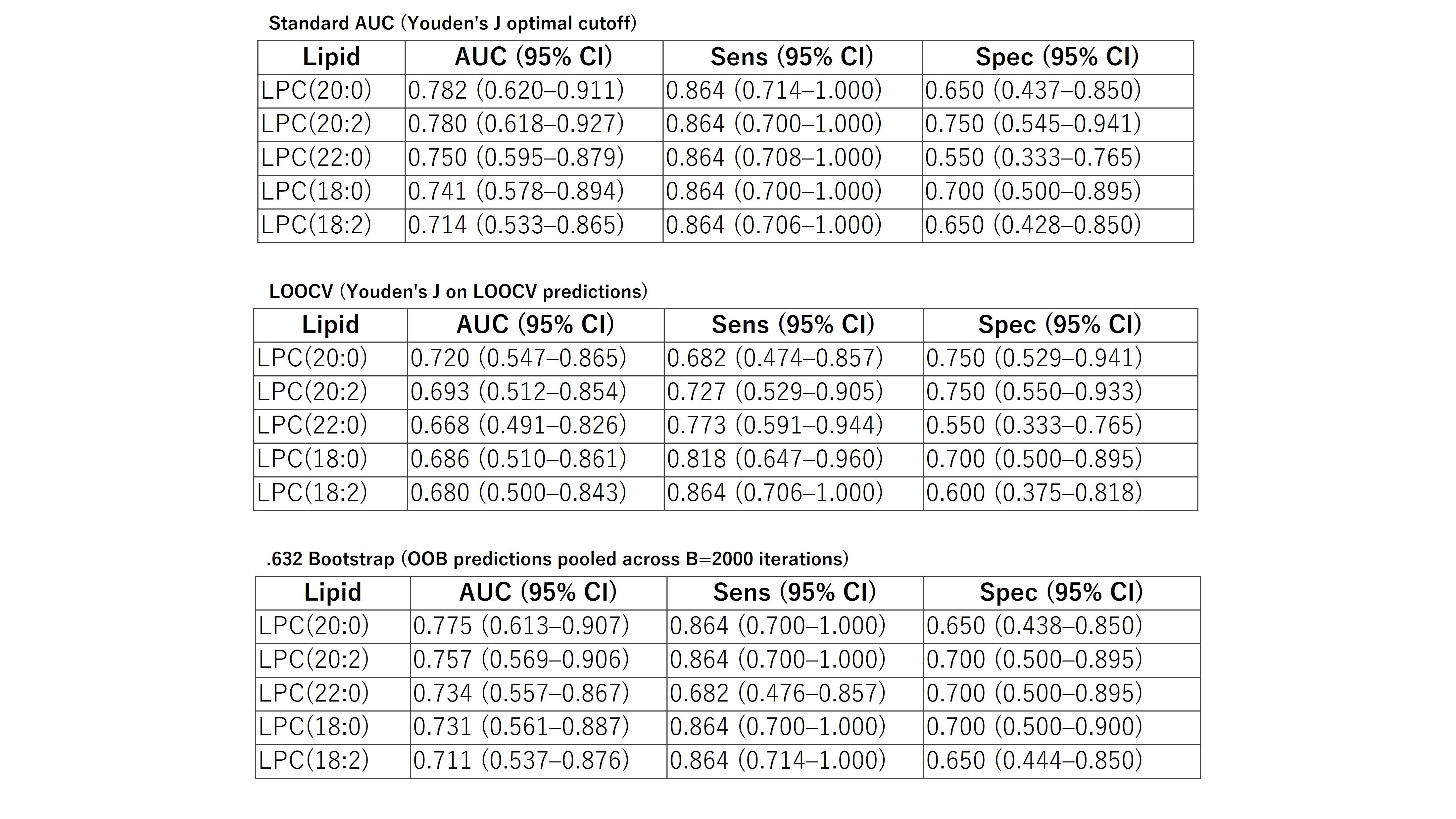

Supplement: Supplementary Figure 2 — Standard AUC values were calculated using all samples (n = 42), with sensitivity and specificity determined at the Youden’s J optimal cutoff. Ninety-five percent confidence intervals were estimated by bootstrap resampling (B = 2,000 iterations). Cross-validation performance was assessed by leave-one-out cross-validation (LOOCV), with 95% CI estimated by bootstrap resampling of LOOCV predictions. The.632 bootstrap AUC was calculated as a bias-corrected estimate of predictive performance. AUC, area under the receiver operating characteristic curve; CI, confidence interval; EV, extracellular vesicle; LPC, lysophosphatidylcholine; LOOCV, leave-one-out cross-validation; NPV, negative predictive value; PPV, positive predictive value; Sens, sensitivity; Spec, specificity. [file Image2.jpeg]

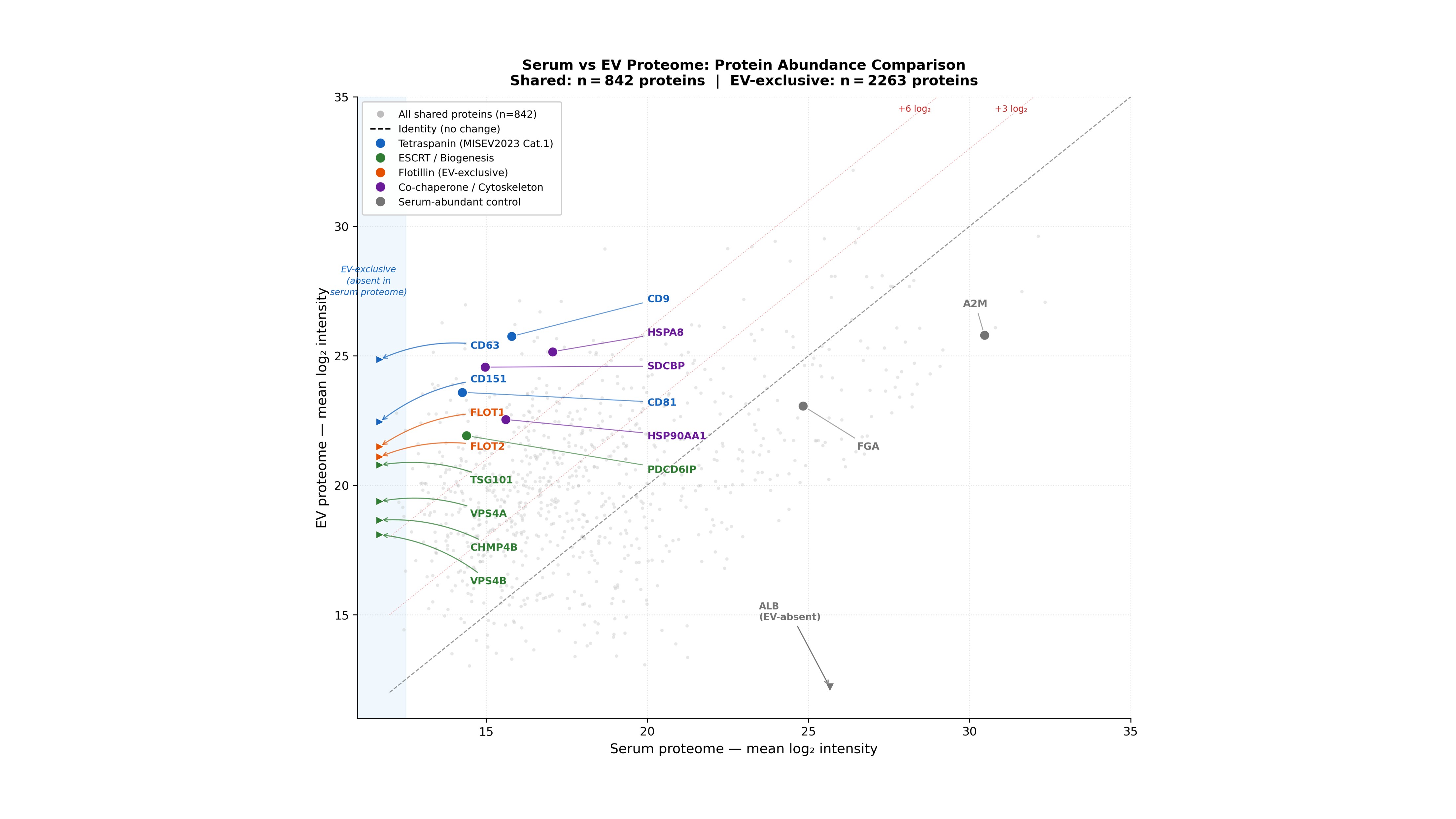

Supplement: Supplementary Figure 3 — EV proteome quality assessment based on protein marker detection. Scatter plot comparing mean log2 protein intensity between the serum proteome and EV proteome across all samples (n = 42). Gray points represent all proteins detected in both fractions. Known EV-associated markers are highlighted: tetraspanins (CD9, CD63, CD81, CD151; blue), ESCRT/biogenesis-associated proteins (TSG101, ALIX/PDCD6IP, VPS4A, VPS4B, CHMP4B; green), flotillins (FLOT1, FLOT2; orange), and co-chaperones (HSPA8, HSP90AA1, SDCBP; purple). Proteins detected exclusively in the EV fraction (EV-exclusive) are indicated along the left shaded region. Serum-abundant proteins (ALB, A2M, FGA) serve as reference controls. Dashed lines indicate the identity line (no difference) and +3/+6 log2 enrichment thresholds. ALB, albumin; ESCRT, endosomal sorting complexes required for transport; EV, extracellular vesicle. [file Image3.jpeg]

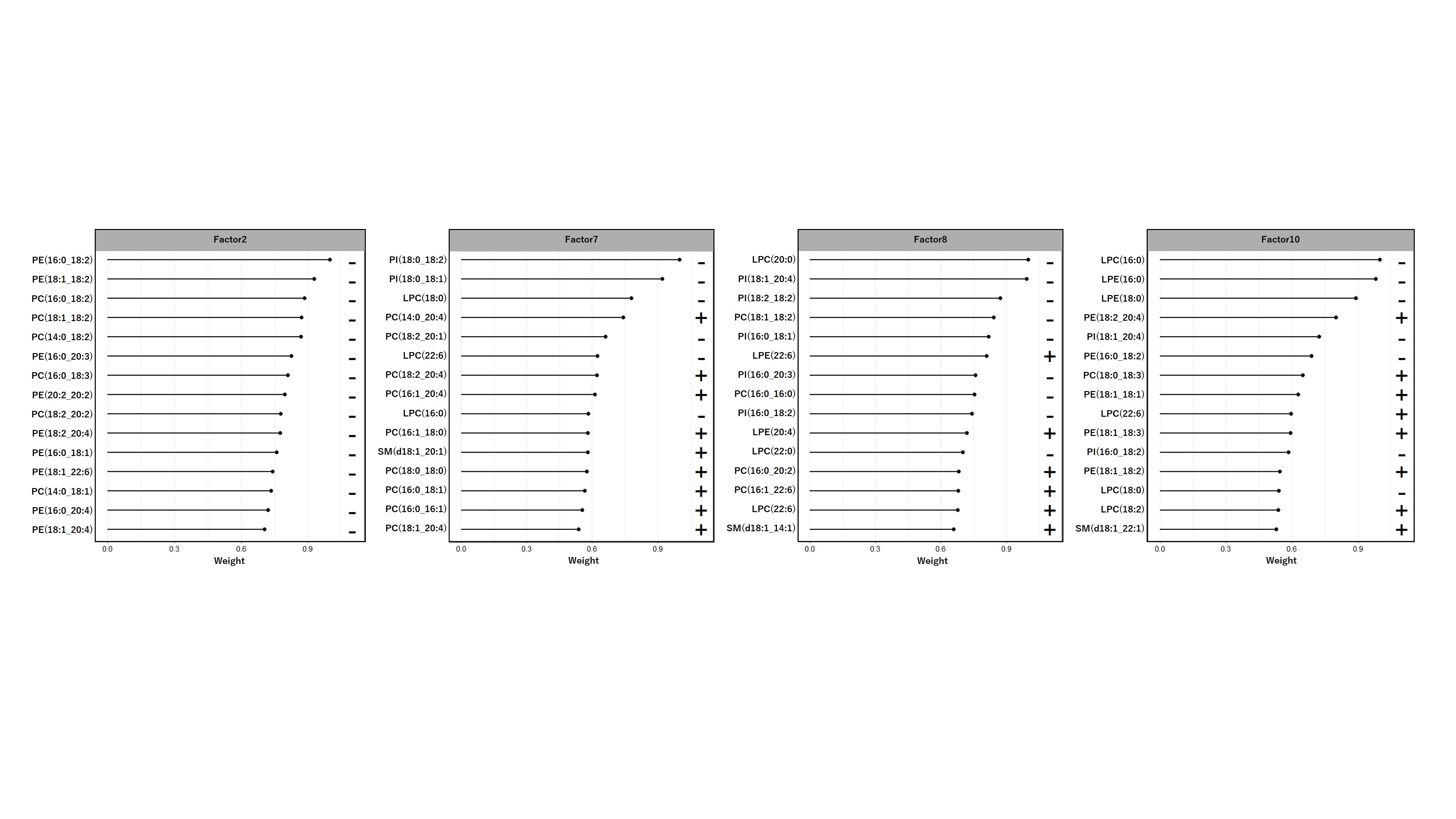

Supplement: Supplementary Figure 4 — Loading plots of MOFA2 factors 2, 7, 8, and 10, which showed significant differences between Responders and Non-Responders. MOFA, multi-omics factor analysis. [file Image4.jpeg]

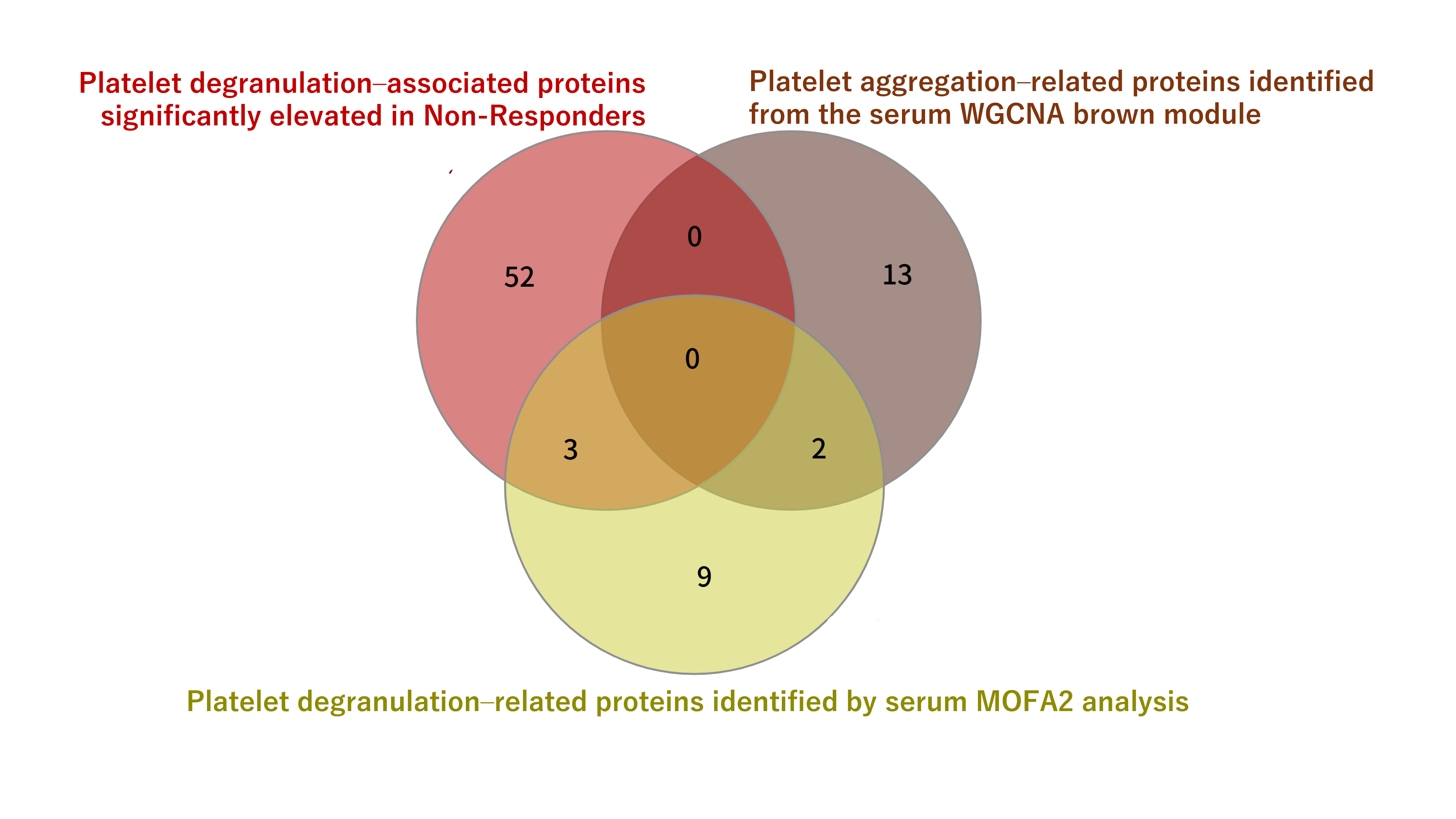

Supplement: Supplementary Figure 5 — Venn diagram depicting the intersection of three protein sets: (1) Platelet degranulation proteins enriched in EVs from Non-Responders, (2) LPC-correlated platelet aggregation-related proteins (WGCNA), and (3) LPC-associated platelet degranulation-related proteins (MOFA2). [file Image5.png]
